# An Exposition of the Symptoms, Essential Nature, and Treatment of Neuropathy, or Nervousness

**Published:** 1837-10

**Authors:** 


### Art. XII.

*An Exposition of the Symptoms, essential Nature, and Treatment of Neuropathy, or Nervousness.* By James Manby Gully, m.d. &c.—*London*, 1837. 8vo. pp. 192.

Mr. Shandy's anxiety to secure for his offspring all the benefits derivable from a well-chosen name, has its parallel in the solicitude which authors manifest to usher their books into the world under the auspices of engaging titles. If such was Dr. Gully's feeling when he composed, or rather, perhaps, translated, the title-page of his work, we suspect that the emotion prevented the calm exercise of the judgment. The etymologist would anticipate that nothing short of the whole pathology of the nerves or nervous system would be included under the new term, if the author had not himself presented the synonyme.

We are informed in the preface that, although much attention has been paid by various writers to hypochondriasis, they have overlooked the fact that this disease is only a more intense degree of nervousness. As they have failed to discern, even in hypochondriasis, the connexion between irritation of the viscera and that of the cerebro-spinal axis, the author thinks it is not wonderful that the morbid sympathy between the two systems should have escaped them in the "milder degree" of the affection. The want of correct views upon this subject is attributed to the fact that "the majority of physiologists, in this country, at least," regard the ganglionic system as "an unmeaning appendix" to the cerebro-spinal. We do not pretend to an acquaintance with the opinions of the actual "majority," but of those who, in the present day, have published their views upon this subject, we are aware of scarcely any who deserve the author's reproach.

The first section of the work treats of the symptoms of Neuropathy, arranged under four heads: 1. Sensations; 2. Movements; 3. Disorders of the internal or organic functions; 4. Mental disorders. Dr. Gully is of opinion that cases of Neuropathy may be distributed into two great classes,—first, those in which "visceral and motor symptoms" predominate; second, those characterized by prevalence of mental disorder; the former belonging to what he calls the minor degree of the malady, the latter to its more intense form, or hypochondriasis.

We cannot imagine what the author's meaning can be in the following passage, with which the section on the Essential Nature of Neuropathy commences:

"The minor degree of neuropathy, the illustrations and the resumé of the symptoms of which have been given, does not appear to have engaged the attention of any writer with whom I am acquainted." (P. 78.)

We have met with little or nothing in the said illustrations that we can imagine to be unfamiliar to one who has read our best systematic works upon Medicine, not to mention the monographs of Cheyne, Whytt, Georget, Louyer-Villermay, Falret, and others, or that vast repertory of curious facts and doctrines, old Burton's Anatomy of Melancholy. It is true that the affections in question are not described as constituting a disease called "Neuropathy, or Nervousness," but they are copiously treated of as disorders to which persons of a particular habit, whether natural or acquired, are liable, and even separately from hypochondriacal and hysterical affections. We think it must be pretty generally understood that nervous affections are derangements of sensation and motion, unconnected with vascular or organic changes, and such as may befal any part of the body where those functions are performed; that, when conjoined with a particular condition of mind, they are represented by the term Hypochondriasis; and that occurring in certain groups in the female subject, they constitute Hysteria. If Dr. G. thought it requisite to urge a greater degree of attention to the pure neuroses, he might have employed himself very profitably in describing their true characters, in contradistinction to the phlegmasiæ and to nutritive and secretory diseases. But this, we apprehend, was not his object; he wished rather to elucidate the nature of a particular group of nervous derangements, to which he has unfortunately applied a designation far too comprehensive. Nervousness, in its usual acceptation, ought to be treated of as a diathesis, not as a disease; but, if it must be considered an actual morbid condition instead of a predisposition, we cannot see the propriety of investing it with a speciality. Viewed in reference to symptoms, its character is *generic:* pathologically, it is an element which may enter into the composition of any malady; for, being essentially a derangement of function in one of the primary tissues, it may own no limits but those of the whole body.

The "essential nature of Neuropathy" receives a very lengthened discussion.

"The opinion," says Dr. G., "which appears to me the most consonant with facts, and the best established by reasoning from them, may be stated thus:—That, in the minor degree of neuropathy, or simple nervousness, the ganglionic matter distributed about the epigastrium is the only permanently diseased point; while, in the more intense degree, disorder of the brain is added to that of the ganglionic matter alluded to, and partially assists in the maintenance of the symptoms." (P. 82.)

The proof of this theory is entered upon by laying down no fewer than eight physiological propositions,—postulates we should rather call them, —not that we are inclined to grant them, nor that, if granted, they can reasonably be urged as claims on our assent to the author's inferences. The first two are these:

"1. The phenomena of life in general are the result of the operation of causes on the irritability of the body.

"2. This irritability is represented by the ganglionic system of nerves as distributed, together with the blood-vessels, to all the tissues of the body." (P. 83.)

Upon the first of these it is needless to dwell, since, if it means any thing, it is resolvable into the truism that all vital actions are the effects of agents upon vitality; for the term irritability is used in the sense of a susceptibility of being so affected by certain agents that certain actions which constitute life are the result, or, in other words, as signifying the fundamental property of life. In the second proposition, we do not exactly understand what is meant by the phrase "represented by," though we think it probable that the author intends only to say that the property is conferred by, or is resident in the ganglionic system. Great authorities are cited in favour of this opinion, though we suspect that the majority of them would be found, upon examination, to go no further than the belief that the function of the ganglionic nerves is exclusively devoted to the processes of organic life; a notion very different from the former. A great number of interesting facts have been collected in favour of the presumption that ganglionic nerves take a very important part in organic actions; but certainly, till they are shown to exist in vegetables, we cannot admit their indispensableness to life; and, even were this evidence forthcoming, it would not prove them to confer vitality; because this property would still be only predicable of the whole organism, whereof the ganglionic nerves form but a part. The three next propositions are as follow:

"3. The grand result of the action of causes on the blood-vessels thus supplied with ganglionic matter is nutrition. 4. All the functions, and even the most sudden changes in them, imply a change in the nutrition of the tissues and organs destined to perform them. 5. There is therefore a change effected in any tissue or organ, the irritability of which, as represented by the ganglionic tissue in it, is acted on by ordinary or extraordinary stimulants." (P. 83.)

Though nutrition must certainly be regarded as the primary action of living bodies, (whether dependent on ganglionic nerves or not,) we do not profess to know the grounds for the dogma in the fourth of these propositions. The function, no doubt, implies the nutrition of the organ destined to perform it; and to a change of the function there may perhaps sometimes be said to be a corresponding change in the nutrition, though not always, since an organ may discharge its office differently at different times, merely from a difference in the materials upon which it has to work, and without any alteration in its own substance. But why the ordinary function should imply a change, or rather a perpetual series of changes, in "the tissue or organ," is to us incomprehensible.

The next proposition is curious.

"6. The last proposition applies to the cerebro-spinal nervous matter, and the result is sensation, succeeded by passion or instinct, thought, and volition.

In other words, "the irritability of the cerebro-spinal nervous matter," "as represented by the ganglionic tissue in it," having been "acted on by ordinary or extraordinary stimulants," a change is effected in that matter, and "the result is sensation," &c. Is it unreasonable to ask for a little more proof than is implied in the "therefore" which ushers in the proposition to which the sixth is a pendant? The two concluding propositions are these:

"7. These phenomena of brain-matter act as indirect stimulants to the irritability of other parts, and therefore play upon the ganglionic nervous system distributed to the viscera.

"8. Impressions made on the viscera, and more especially those largely supplied with ganglionic nervous matter, are reflected on the brain matter, and modify its functions." (P. 85.)

The illustration of these statements occupies several pages, an accurate digest of which we can hardly promise; for we are not quite sure that we have always thoroughly understood the author, whose forte does not appear to consist in arranging his thoughts in the most logical order, or in clothing them in the most lucid phraseology. So far, however, as we could follow the argument, it runs thus: "Impressions commencing in the external senses and the brain are necessarily reflected upon the viscera, and therefrom derive their character and intensity;" as, for instance, the sight or smell of food is agreeable or otherwise, and is judged of by the brain according to the "responsive echo" from the viscera. The principal function of the brain is to receive and transmit impressions from one part to another without intermission. In this transmission of impressions, "the brain is ever playing upon the viscera and receiving stimulation from them," even during consciousness and intellectual processes, since we know that the latter may be disturbed by visceral sensations. But, if impressions originating in the brain are thus communicated to the viscera, it is easy to believe that impressions on the latter must influence the cerebral actions; a fact exemplified in the stimulating effects of food on the voluntary muscles. The author then proceeds to state that the conductors of impressions between the cerebral and epigastric centres are ganglionic nerves; and that, inasmuch as the brain receives a larger proportion of blood than any other organ, it must, according to the second proposition, enjoy "a proportionate degree of irritability, and therefore of ganglionic matter, in its tissue." Although, then, the great sympathetic is the more concentrated collection of ganglionic matter, the brain approaches nearer to it in this respect than any other organ; and, as these collections of "irritable matter" are continuous, the sympathies between them are inevitable. The mutual communications are "transfers of irritation;" and, as functions imply changes of nutrition, it must follow that the nutritive irritation of the two centres is reciprocally modified.

After this physiological proem, the author proceeds to illustrate the operation of such agents as disturb the harmony of the two nervous systems. Thus, in excessive mental application, the brain becomes a focus of irritation to the viscera, which may react upon it to the point of establishing the "major degree of neuropathy." A similar process is traced in the effect of strong passions, and of violent muscular exertion, as well as a converse operation of ennui, in withholding from the viscera the cerebral stimulation to which they have been habituated. The influence exerted by excitants of the internal sentient surface, particularly by purgatives, is next illustrated; and then the effect of excessive menstruation and lactation, which are shewn to act by transference of "long-continued irritation" of the womb and the breasts to the central nervous nutritive system, and thence to the brain.

The author considers his theory to be "as well entitled to credit as any theory can be which rests upon presumptive evidence alone." We suspect that his conviction is in a great measure assignable to the equivocal meaning of the term irritation. Were the latter used as synonymous with disturbed function, few would be inclined to deny that the early symptoms of such cases as Dr. G. describes belong to the nerves of the viscera; they are, indeed, usually called nervous sensations and nervous derangements, &c., to shew that they are idiopathic, or not secondary to vascular and molecular changes. The current opinion, again, respecting these sensations would readily concede them to be often, in the first instance, excited by cerebral disorder of some kind, and in their turn to react upon the latter; whether by means of what Dr. G. denominates organic reverberations, or by what is commonly called sympathy, is a matter of no great consequence. But Dr. G. sets out with adopting the strict physiological meaning of irritation, as synonymous with vital action, which, of course, comprehends every kind of function. Now, if the vital actions of the viscera were increased, there must be an augmentation of their nutrition, of their movements, of their secreting and absorbing functions, &c.; but are such effects to be traced in the symptoms which the author has himself detailed, the anorexia, the impaired digestion, the constipation, the sense of sinking, &c.? If the brain of the over-tasked student communicates its increased irritation (physiological) to the stomach, the muscular fibres of the latter should be in a high state of nutrition and contractile power; the gastric juice should be poured out in abundance, and of the best quality; and the nerves should reflect, either no sensations at all (unconsciousness of digestion being the most healthy condition,) or none but agreeable ones; while the fact is the reverse of all this.

Dr. Gully's explanation of the effect of suppressed menstruation, or of the drying up of old ulcers, exhibits alike confusion either in his apprehension or in his employment of the term irritation. Having been told that, in a state of health, the organs exchange healthy irritations, we are prepared to hear that suppression of the uterine function occasions disturbance merely because there is a failure in the supply of the normal irritation: instead of this, however, the author tells us "that the womb becomes a source of irritation to other parts," and that "the reverberation would be on the great sympathetic." We should have thought that, according to his views, there would be no reverberation at all, the want of which was the cause of the remote derangement. In like manner, when explaining the effect of suppressed ulcers, he remarks, that, inasmuch as these "established forms of nutritive irritation" have become necessary to other parts, "*when suppressed, they irritate the centre of all irritability!*"

But do we deny that affections of the viscera communicate with each other by means of ganglionic nerves, or that the latter are the media of intercourse between the viscera and the cerebro-spinal system? Certainly not; for, though absolute proof may be wanting, we have strong presumptions that ganglionic nerves do maintain the harmony of the organic functions, and that they convey the influence of mental and moral changes to the viscera, and return impressions from these organs to some part of the cerebro-spinal system. It is, therefore, probable that ganglionic nerves are deeply involved in some of the disorders which constitute Dr. G.'s milder neuropathy, and that, when those which constitute the severer form occur subsequently to the visceral derangements, the same nerves may bear a part in the morbid communication. But we deny altogether that affections of the brain must necessarily be preceded by ganglionic disorder; firstly, because it has not been proved that ganglionic matter is essential to vital action; 2dly, because it is a mere conjecture that ganglionic nerves accompany the vessels of the brain in such numbers as to form a large collection of ganglionic matter in that organ; and, 3dly, because many serious diseases of the brain, (diseases which must inevitably implicate ganglionic matter, if this is really essential to vital action,) may occur without producing any "organic reverberations" from the viscera. Had Dr. G. contented himself with indicating the ganglionic system as the probable seat of certain neurotic affections of the viscera, often called nervous, hysterical, and hypochondriacal, we should only have been reminded of an opinion very common among pathologists of the present day; but, by connecting this view with certain hypothetical notions respecting physiological irritation, he has mystified rather than elucidated his subject.

In the remarks upon "Constitutional Nervousness," we find a very clear and faithful representation of the principal features of the nervous diathesis; and this we consider the best executed part of the work. We cannot speak as favorably of the theoretical explanation of that condition. The author deems it "highly probable that congenital or constitutional nervousness consists in an unusual development of the visceral ganglionic system, and consequent unusual susceptibility to impressions from the brain."

In the therapeutical section we hoped to have been compensated for time and attention spent upon profitless speculations, by some valuable additions to our remedial resources. But here, again, the chief space is occupied by fanciful conjectures about the operation of remedies which practitioners in general are content to use from the experience of centuries in their favour; while, in the directions for their use, we meet with cautions which should be familiar to every student. The hygienic treatment recommended by Dr. G. appears to us upon the whole very judicious, and corresponds with the rules given by our best-approved writers on diet, management of the skin, exercise, &c. But it is to be regretted that even these plain matters should be mystified by the strange interpretations with which the author thinks it worth while to overlay them.

